# Post‐Traumatic Stress Disorder (PTSD) Among Algerian Cancer Patients: Validity of the Arabic DSM‐5 PTSD Checklist (PCL‐5) and Associated Factors

**DOI:** 10.1002/pon.70109

**Published:** 2025-02-21

**Authors:** Aiche Sabah, Fethi Hammadi, Chiu‐Hsiang Lee, Musheer A. Aljaberi, Monique van Dijk, Chung‐Ying Lin, Mark D. Griffiths

**Affiliations:** ^1^ Faculty of Human and Social Sciences Hassiba Benbouali University of Chlef Chlef Algeria; ^2^ Department of Nursing Chung Shan Medical University Taichung Taiwan; ^3^ Department of Nursing Chung Shan Medical University Hospital Taichung Taiwan; ^4^ Department of Internal Medicine Section Nursing Science Erasmus University Medical Center (Erasmus MC) Rotterdam The Netherlands; ^5^ Institute of Allied Health Sciences College of Medicine National Cheng Kung University Tainan Taiwan; ^6^ Biostatistics Consulting Center National Cheng Kung University Hospital, College of Medicine, National Cheng Kung University Tainan Taiwan; ^7^ College of Nursing Kaohsiung Medical University Kaohsiung Taiwan; ^8^ International Gaming Research Unit, Psychology Department Nottingham Trent University Nottingham UK

**Keywords:** cancer patients, DSM‐5 PTSD Checklist (PCL‐5), post‐traumatic stress disorder (PTSD), psycho‐oncology, psychological impact of cancer, psychometric validation, stress, trauma

## Abstract

**Background:**

Cancer patients are at risk of post‐traumatic stress disorder (PTSD) throughout their treatment journey due to serious challenges (e.g., complex surgical interventions, severe pain, and side effects from chemotherapy or radiation therapy). This may worsen patients’ health and negatively impact their overall treatment journey and well‐being. However, this area remains understudied in Algeria. Therefore, there is a need to understand the levels of PTSD symptoms and the associated factors among Algerian patients. To ensure accurate assessment and diagnosis, a validation study was conducted to confirm that the PTSD measurement instrument used was culturally appropriate for the Algerian context. The study’s main objectives were to (i) determine the prevalence of PTSD among cancer patients in Algeria, (ii) validate the Arabic DSM‐5 PTSD Checklist (PCL‐5) among Algerian cancer patients, and (iii) explore the associations between demographic and clinical factors and PTSD among this population.

**Methods:**

The present study was cross‐sectional and comprised 370 cancer patients. All participants were patients receiving treatment in oncology wards across various hospitals. All participants completed the PCL‐5. Confirmatory factor analysis (CFA) was used to examine the factor structure of the PCL‐5.

**Results:**

The PCL‐5 was found to have a four‐factor structure. Out of the 370 participants, 154 (41.6%) had PCL‐5 scores below the cutoff of 31, indicating lower levels of PTSD symptoms; 216 participants (58.4%) had scores above this threshold, suggesting a likelihood of PTSD. Moreover, PTSD was significantly associated with patients' low educational attainment and more advanced stages of their disease.

**Conclusions:**

The findings indicated moderate to high PTSD symptoms among cancer patients in Algeria. Moreover, the Arabic PCL‐5 demonstrated good psychometric properties confirming that it is a reliable and valid tool for assessing PTSD symptoms among Algerian cancer patients.

## 1 Introduction

Cancer is a major global public health challenge, affecting individuals in both developed and developing countries [1, 2, 3]. It is the second leading cause of death worldwide, with nearly 20 million new cases and 9.7 million deaths reported in 2022. Incidence rates vary significantly by region, ranging from over 500 per 100,000 in Australia/New Zealand to under 100 per 100,000 in parts of western Africa. By 2050, cancer cases could reach 35 million [4]. Low‐ and middle‐income countries (LMICs) face a disproportionate burden, accounting for 70% of global cancer deaths [5], largely due to aging populations, increased cancer risk behaviors, and limited access to effective treatments [6, 7]. This growing cancer burden underscores the urgent need for improved prevention and care strategies worldwide.

A cancer diagnosis is a profoundly distressing experience, often perceived as life‐threatening [8]. This distress can lead to various mental health issues, including depression, anxiety, and existential distress, ultimately impacting pain control, treatment compliance, and patients’ willingness to pursue therapy [9, 10]. Post‐traumatic stress disorder (PTSD) is a major consequence for approximately 10%–20% of cancer patients, mostly from the intensity of treatment. For example, Chan et al. [11] reported that 21.7% of cancer patients had PTSD 6 months after diagnosis. Similarly, Wu et al. [12] reported that 9.6% of breast cancer patients suffered from symptoms indicative of PTSD symptoms. They also reported that the risk of PTSD was related to (i) age (with younger patients being at higher risk than older patients), and (ii) having recently stopped treatment.

Psychological factors, including trauma, grief, and depression, have been identified as critical contributors to the development of breast and lung cancer before diagnosis, underscoring their relevance to prevention and intervention strategies [13]. Similarly, psychological characteristics such as individuals’ negative appraisals of their experience, avoidance‐based coping mechanisms, and limited social support are significantly associated with heightened PTSD symptomatology among bone marrow transplant patients [14].

Cancer‐related PTSD (CR‐PTSD) affects a minority of survivors, with estimates ranging from 0% to 22% based on structured interviews and as high as 55% in self‐report studies [8, 15, 16]. The impact of PTSD on quality of life can be profound, affecting psychosocial functioning and treatment adherence [17]. Key risk factors include the severity of the cancer diagnosis, treatment‐related stress, and pre‐existing mental health conditions [18]. Given the symptoms outlined in the DSM‐5, it is crucial for healthcare professionals to recognize and screen for PTSD because early intervention can significantly improve patient outcomes [19]. A holistic approach that integrates psychological support into cancer care is essential for addressing the complex needs of these patients [20].

The discrepancy in reported PTSD levels among cancer patients highlights the importance of assessment methods. Research indicates that advanced‐stage cancer patients tend to show higher PTSD levels, with younger patients and those who have recently completed treatment also exhibiting a higher PTSD prevalence [8, 15]. Additionally, female cancer patients generally report more severe PTSD symptoms compared to males [21, 22]. PTSD among cancer patients is often associated with other conditions, such as anxiety and depression, with significant associations between PTSD symptoms and scores on the Hospital Anxiety and Depression Scale (HADS) [21, 22].

Research has increasingly acknowledged the potential for cancer diagnosis and its treatment to induce PTSD [23, 24]. Therefore, screening for PTSD symptoms among cancer patients is essential to identify individuals who need support and intervention [22]. Among different assessment tools, the Post‐traumatic Stress Disorder Checklist for DSM‐5 (PCL‐5) is a widely regarded self‐report measure designed to assess PTSD symptoms based on DSM‐5 criteria. Its validity and reliability have been established in various cultural contexts [25, 26, 27]. Similarly, research involving war‐affected Kurdish and Arab populations in Iraq found the PCL‐5 to have high internal consistency and good convergent validity compared to experts' ratings. However, structural validity was not determined [28]. However, to date, there is no validated Arabic version of the PCL‐5 specifically for cancer patients in Algeria.

The PCL‐5 has demonstrated strong psychometric properties across various populations. For example, Hoeboer et al. [29] validated the Dutch version among trauma‐exposed adults, finding excellent diagnostic accuracy and recommending a cutoff score of 22 for screening. Similarly, Di Tella et al. [30] confirmed the Italian version's seven‐factor structure and strong concurrent validity. Other studies, such as Cernovsky et al. [31] with car accident survivors, have further validated the instrument's criterion and convergent validity. DuHamel et al. [32] applied it successfully among cancer survivors, while Smith et al. [33] corroborated its usefulness among cancer survivors with a strong association with the PC‐PTSD‐5. These studies collectively affirm the PCL‐5's reliability and validity across diverse populations.

Concerns regarding the validity and reliability of PTSD assessment instruments are heightened by cultural differences, as well as demographic and clinical factors that can affect assessment outcomes, emphasizing the need for psychometric evidence among specific populations, such as cancer survivors. Research has identified that various demographic and clinical factors contribute to the development of PTSD among cancer survivors, including lower educational levels, being unmarried, being unemployed, and economic hardship [34, 35]. While some studies indicate no significant relationship between PTSD severity and cancer stage [36], others suggest otherwise [35].

Despite extensive global research, data on PTSD levels among cancer patients in Algeria remains scarce, partly due to the lack of validated assessment instruments such as the PCL‐5. The present study addressed this significant research gap by validating the Arabic version of the PCL‐5 and examining PTSD prevalence and its correlates among this population. The findings aimed to provide psychometric evidence and practical insights that can inform culturally appropriate interventions. The following three research questions (RQs) guided the study: (i) what is the prevalence of PTSD among cancer patients in Algeria? (RQ1), (ii) is the Arabic version of the PCL‐5 a valid and reliable instrument for assessing PTSD among cancer patients, as demonstrated through confirmatory factor analysis (CFA)? (RQ2), and (iii) how are PTSD levels associated with demographic, clinical, and social factors such as education, economic status, family status, diagnosis, disease stage, surgery, gender, and age among Algerian cancer patients? (RQ3).

## 2 Methods

### 2.1 Study Design and Participants

The present cross‐sectional study was conducted over a two‐year period from April 2022 to July 2023 among cancer patients receiving treatment for various types of cancer at hospitals in Chlef and Oran (western Algeria). The sample was selected based on the inclusion and exclusion criteria with cases under follow‐up at the targeted hospitals, with assistance from the clinical psychologist in the department. Socio‐demographic and clinical variables were verified directly with patients during survey administration. The inclusion criteria comprised cancer patients receiving regular treatment in Oran and Chlef hospitals, those able to read and write for survey comprehension, individuals who voluntarily consented to participate, and patients without physical or psychological impairments that could hinder survey responses. Participants were also required to be in stable follow‐up stages of treatment. The exclusion criteria included patients who were illiterate, individuals with severe psychological disorders impairing concentration or interaction, those with other chronic illnesses that could confound results, and patients who declined participation. Additional exclusions applied to patients receiving treatment outside Oran and Chlef, those with unstable or emergency health conditions, individuals with severe physical limitations (e.g., significant visual impairment), and patients with communication barriers (e.g., hearing impairments or lack of proficiency in the study language).

Participants were recruited using convenience sampling and completed a “paper‐and‐pencil” survey at the hospital, assisted by the researchers after consulting with the psychologist in the cancer ward. Written informed consent was obtained before data collection, and the study's objectives were explained to the patients. A research team member clarified any unclear items for the participants.

### 2.2 Measure

The Post‐traumatic Stress Disorder Checklist (PCL) is a widely used self‐report measure that assesses the severity of PTSD symptoms. It is designed to evaluate symptom severity over the past month and provides a provisional PTSD diagnosis based on DSM criteria [37]. The PCL was revised to reflect DSM‐5 changes to the PTSD criteria, resulting in the development of the PCL‐5 [38, 39]. The National Center for PTSD in the U.S. Department of Veterans Affairs [40] defines the PCL‐5 as a self‐report tool that assesses 20 PTSD symptoms as outlined in the DSM‐5. Individuals rate their symptoms based on five response options: 0 (*not at all*), 1 (*a little bit*), 2 (*moderately*), 3 (*quite a bit*), and 4 (*extremely*). These symptoms are organized into four clusters aligning with the core criteria of PTSD in the DSM‐5: intrusion (Items 1–5), avoidance (Items 6–7), negative alterations in cognitions and moods (Items 8–14), and alterations in arousal and reactivity (Items 15–20).

The total symptom severity score for PTSD is calculated by summing the scores of the 20 items, with a possible range from 0 to 80. To make a provisional PTSD diagnosis, any item rated 2 (*Moderately*) or higher is considered a symptom. According to DSM‐5 criteria, a diagnosis requires at least one symptom from Category B (Items 1–5), one from Category C (Items 6–7), two from Category D (items 8–14), and two from Category E (items 15–20). Preliminary research suggests that a cutoff score between 31 and 33 may indicate probable PTSD [40]. The PCL‐5 has demonstrated strong psychometric properties, including internal consistency (*α* = 0.94), test‐retest reliability (*r* = 0.82), and convergent validity with the Post‐traumatic Stress Disorder Checklist (*r* = 0.85), Post‐traumatic Stress Diagnostic Scale (*r* = 0.85), and Detailed Assessment of Post‐traumatic Stress (*r* = 0.85); and discriminant validity with Antisocial Personality Features and Mania (*r*‐values = 0.31 to 0.60) [37].

The PCL‐5 is used in clinical settings and research, allowing patients to complete it before sessions or while waiting [39]. It has been validated across different languages, including German [41], Brazilian [42], Turkish [43], Dutch [29], Italian [30], and Arabic [44], and has been applied to cancer patients and motor vehicle accident survivors, showing suitability for these populations [31, 33]. The Arabic version, translated by Ibrahim et al. [28], was validated in the Kurdistan region of Iraq, demonstrating high internal consistency (*α* = 0.85) and adequate convergent validity, with significant correlations between PTSD symptoms and traumatic event exposure, further supported by comparison with expert assessments.

### 2.3 Statistical Analysis

The data were analyzed using SPSS 26 and AMOS 24. The mean and standard deviation (SD) were applied for normally distributed continuous variables, and the median with interquartile range for non‐normally distributed variables. Skewness and kurtosis were calculated to assess the distribution shape and peak of responses. Skewness values between −1 and +1 are considered excellent, while kurtosis values should fall within the range of −2 to +2 [45, 46]. There were no missing data. To estimate the level of PTSD, a PCL‐5 cutoff score between 31–33 was chosen based on its effectiveness across various samples. Several methods exist for determining cutoff scores for the PCL‐5, but the optimal range is between 31 and 33 [40]. Previous research, including studies on cancer patients, has suggested that a cutoff score of 31 is appropriate for identifying probable PTSD [47, 48, 49]. Therefore, a score of 31 was selected for the present study which was used to estimate the prevalence of PTSD among cancer patients in Algeria (to answer RQ1 in the present study).

CFA was conducted to verify the four‐factor structure of the PCL‐5 (to answer RQ2 in the present study). To examine model fit quality, the following indicators were used: chi‐square/df between 1 and 3, comparative fit index (CFI) and Tucker‐Lewis index (TLI) above 0.90, and root mean square error of approximation (RMSEA) and standardized root mean square residual (SRMR) less than 0.08 [50, 51, 52, 53]. The reliability of the PCL‐5 was assessed through internal consistency measured by Cronbach's alpha (> 0.7 indicating satisfactory), along with composite reliability (CR; > 0.7 indicating satisfactory), average variance extracted (AVE; > 0.5 indicating satisfactory), and MaxR(H) (> 0.7 indicating satisfactory), which are essential for evaluating the model’s validity in CFA [54, 55]. Complementary analyses (including independent samples *t*‐tests and analysis of variance [ANOVA] with least significant difference [LSD] post hoc tests) were conducted to examine the relationships between various demographic variables and PTSD. These analyses aimed to explore potential differences in PTSD symptoms across different demographic groups (to answer RQ3 in the present study). Additionally, multiple regression analysis was conducted to examine the association between demographic and clinical characteristics and PTSD among cancer patients. This standard multiple regression, known as the Enter method, involves the simultaneous entry of all independent variables. It considered the following variables as possible explanatory factors for PTSD: educational level, economic status, marital status, first diagnosis, disease stage, cancer‐related surgery, chemotherapy duration, and family medical history of cancer (also to answer RQ3 in the present study).

## 3 Results

### 3.1 Descriptive Statistics and Statistical Differences by Demographic and Medical Variables

Table 1 presents the demographic and clinical characteristics of the participating cancer patients who took part in the study (*N* = 370; 118 males [31.9%] and 252 females [68.1%]). The average age of patients was 51.09 years (SD = 14.25). The majority of the participants were initially diagnosed with cancer during the study period (81.6%).

**TABLE 1:** Demographic, medical characteristics, and PTSD scores with statistical differences.

Variable	Category	N	%	Mean	SD	Statistical differences
Gender	Male	118	31.9	38.66	17.11	*t* = 2.14
	Female	252	68.1	34.80	15.77	*p* = 0.03
Educational level	Primary	97	26.2	42.04	16.34	*F* = 6.485
	Secondary	213	57.6	34.27	16.19	*p* < 0.001.
	University	44	11.9	33.13	14.17	
	Other	16	4.3	30.93	14.26	
Economic status	Poor	69	18.6	38.88	15.85	F = 2.174
	Average	270	73.0	35.79	16.48	*p* = 0.115
	Good	31	8.4	31.74	14.81	
Marital status	Single	65	17.6	33.90	15.74	*F* = 0.679
	Married	286	77.3	36.51	16.63	*p* = 0.508
	Divorced	19	5.1	36.00	12.48	
First diagnosis	Yes	302	81.6	36.23	16.23	*t* = 0.496
	No	68	18.4	35.14	16.61	*p* = 0.620
Disease stage of cancer	I	211	57.0	33.59	16.14	*F* = 5.584
	II	121	32.7	40.23	16.05	
	III	31	8.4	38.51	15.02	*p* = 0.001
	IV	7	1.9	26.00	13.85	
Surgery	Yes	228	61.6	35.31	15.91	*t* = −1.072
	No	142	38.4	37.18	16.87	*p* = 0.284
Chemotherapy duration	6 months	156	42.2	35.51	16.72	*F* = 0.157
	1 year	105	28.4	36.79	17.05	
	2 years	45	12.2	36.59	13.65	*p* = 0.925
	3 or more years	64	17.3	35.64	15.92	
Family medical history of cancer	Yes	137	37.0	34.83	16.16	*t* = −1.087
	No	233	63.0	36.73	16.35	*p* = 0.278

Males had significantly higher PTSD scores (*M* = 38.66) than females (*M* = 34.80; SD = 15.78; *t*(368) = 2.134, *p* = 0.033). Moreover, the primary education group had the highest score (*M* = 42.04, SD = 16.35; *F* = 6.485, *p* < 0.001). No significant differences were found in PTSD scores for economic status (*F* = 2.174, *p* = 0.115), marital status (*F* = 0.679, *p* = 0.508), first injury (*t*(368) = 0.496, *p* = 0.620), surgical status (*t*(368) = −1.072, *p* = 0.284), chemotherapy duration (*F* = 0.157, *p* = 0.925), or family medical history (*t*(368) = −1.087, *p* = 0.278). However, disease stages significantly differed in PTSD scores (*F* = 5.584, *p* = 0.001), with post hoc tests identifying differences between stages.

### 3.2 Results of Item Property Analysis: The Prevalence of PTSD Among Cancer Patients in Algeria

Table 2 presents a comprehensive analysis of responses to items evaluating reactions to stressful experiences. The mean values of items ranged from 1.31 to 2.51, reflecting varying symptom intensities. For instance, “Feeling very upset when reminded of the stressful experience” had the highest mean (2.51), indicating significant emotional distress triggered by reminders. Conversely, “Taking too many risks or doing things that could cause you harm” had the lowest mean (1.31), suggesting a lower level of risk‐taking behavior. Table 2 additionally shows that 216 out of 370 (58.4%) participants had probable PTSD.

**TABLE 2:** Descriptive statistics and distribution of the DSM‐5 PTSD checklist (PCL‐5) Items.

Item content	Mean	SD	Skw	Kur	%				
					N	A	M	Q	E
Repeated, disturbing, and unwanted memories of the stressful experience	2.08	1.39	−0.01	−1.31	15.4	24.9	17.3	20.8	21.6
Repeated, disturbing dreams of the stressful experience	1.60	1.338	0.37	−1.06	26.8	25.7	19.7	16.5	11.4
Suddenly feeling or acting as if the stressful experience were actually happening again	1.74	1.26	0.17	−1.03	20.5	24.6	24.6	20.5	9.7
Feeling very upset when something reminds you of a stressful experience	2.51	1.40	−0.46	−1.08	12.2	13.5	20.3	18.9	35.1
Having strong physical reactions when something reminds you of the stressful experience	2.46	1.52	−0.45	−1.31	17.3	13.5	13.2	17.8	38.1
Avoiding memories, thoughts, or feelings related to the stressful experience	1.97	1.32	0.02	−1.13	16.8	22.2	23.8	21.6	15.7
Avoiding external reminders of the stressful experience	1.91	1.35	0.09	−1.21	18.9	24.1	20.0	21.4	15.7
Trouble remembering important parts of the stressful experience	1.59	1.36	0.29	−1.15	30.8	17.8	23.5	16.8	11.1
Having strong negative beliefs about yourself, other people, or the world	1.61	1.42	0.30	−1.29	31.9	20.3	15.4	20.3	12.2
Blaming yourself or someone else for the stressful experience	1.57	1.44	0.29	−1.31	0.3	36.2	12.4	20.8	12.4
Having strong negative feelings such as fear, horror, anger, guilt, or shame	1.84	1.34	0.08	−1.20	21.1	22.2	20.8	23.2	12.7
Loss of interest in activities that you used to enjoy	1.75	1.32	0.11	−1.15	24.3	18.9	24.9	21.1	10.8
Feeling distant or cut off from other people	1.66	1.39	0.27	−1.24	27.8	22.7	17.0	20.3	12.2
Trouble experiencing positive feelings?	1.69	1.40	0.21	−1.23	28.9	17.3	22.4	18.1	13.2
Irritable behavior, angry outbursts, or acting aggressively	1.59	1.42	0.33	−1.25	32.2	19.7	17.3	18.4	12.4
Taking too many risks or doing things that could cause you harm	1.31	1.43	0.68	−0.92	43.8	16.8	16.5	10.8	12.2
Being “superalert” or watchful or on guard	1.70	1.24	0.25	−0.88	20.5	24.9	28.4	16.2	10.0
Feeling jumpy or easily startled	1.61	1.27	0.22	−1.04	26.5	20.5	26.5	18.4	8.1
Having difficulty concentrating	1.81	1.34	0.17	−1.16	21.1	24.3	21.1	20.0	13.5
Trouble falling or staying asleep	2.01	1.43	0.03	−1.33	19.7	21.1	19.7	17.6	21.9
Total score	36.03	16.29	0.04	−0.80	25.7	21.1	21.1	18.9	13.2
Cutoff‐point for probable PTSD	PCL‐5 total score ≥ 31			154 (41.6%)					
	PCL‐5 total score < 31			216 (58.4%)					

Abbreviations: A = a little bit (score 1); E = extremely (score 4); Kur = kurtosis; M = moderately (score 2); N = not at all (score 0); Q = quite a bit (score 3); Skw = skewness.

### 3.3 CFA Findings of the PCL‐5

**FIGURE 1:**
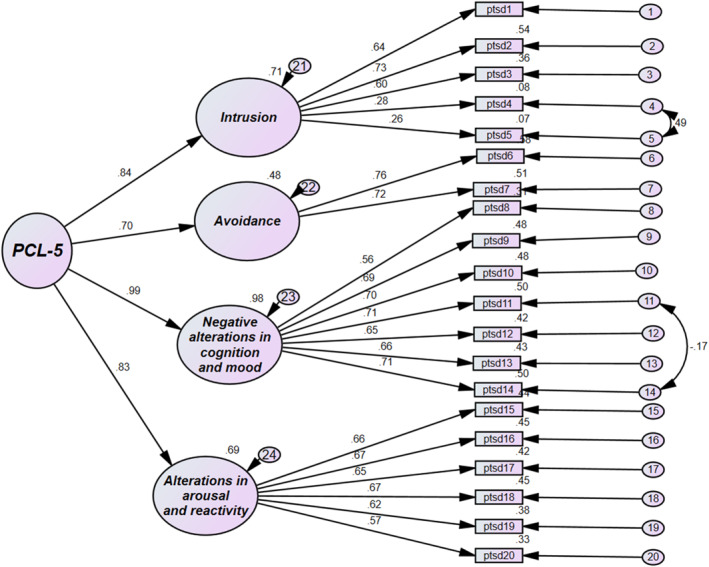
Factor loadings of the DSM‐5 PTSD checklist (PCL‐5).

The results of the fit indices indicated a good model fit for the four‐factor structure: chi‐square/df = 2.084, CFI = 0.931, TLI = 0.92, RMSEA = 0.054, and SRMR = 0.050. Moreover, the second‐order CFA model results indicated significant factor loadings (Figure 1) for the retained items, ranging from 0.26 (Item 5) to 0.76 (Item 6). The factor loadings of the subscales ranged from 0.70 (avoidance) to 0.99 (negative alterations in cognition and mood).

Several key metrics supported the reliability of the PCL‐5 well (CR = 0.908, AVE = 0.715, MaxR(H) = 0.979). Additionally, Cronbach's alpha was 0.903, further confirming the scale's internal consistency. Specific dimensions of the PCL‐5 also demonstrated reliable metrics, with intrusion (*α* = 0.68), avoidance (*α* = 0.67), negative alterations in cognitions and moods (*α* = 0.84), and alterations in arousal and reactivity (*α* = 0.81), underscoring the acceptability of the scale across its various components.

### 3.4 Demographic and Clinical Characteristics Associated With PTSD Among Cancer Patients

The regression model was significant (*F* = 4.056; *p* < 0.001) with an *R* ^2^ of 0.082 (adjusted *R* ^2^ = 0.062). Further analysis of the coefficients, as shown in Table 3, indicated the specific association of each factor to the PCL‐5 scores. Educational level was found to have a significantly negative association with PTSD (*B* = −3.06, SE = 0.77; *p* < 0.001), indicating that higher educational levels are associated with lower levels of PTSD. The stage of disease progression was positively associated with PCL‐5 scores (*B* = 3.05, SE = 1.17; *p* < 0.05), suggesting that more advanced stages of disease are associated with higher levels of PTSD.

**TABLE 3:** Results of the effect of demographic and clinical characteristics on PTSD.

Variables	Unstandardized coefficients		Standardized coefficients	*t*	*p*	95% confidence interval for B	
	B	Std. error	Beta			Lower bound	Upper bound
Educational level (ref: primary)	−3.06	0.77	−0.20	−3.97	< 0.001	−4.57	−1.54
Economic status (ref: poor)	−2.09	1.64	−0.06	−1.27	0.20	−5.33	1.13
Family status (ref: single)	1.42	1.91	0.04	0.74	0.45	−2.33	5.19
First diagnosis (ref: yes)	−2.03	2.20	−0.04	−0.92	0.35	−6.37	2.30
Stage of disease (ref: first)	3.05	1. 17	0.13	2.59	0.01	0.73	5.37
Surgical treatment (ref: yes)	2.14	1.69	0.06	1.26	0.20	−1.20	5.48
Gender (ref: male)	−2.71	1.85	−0.07	−1.46	0.14	−6.35	0.93
Age	0.005	0.06	0.005	0.08	0.93	−0.12	0.13

## 4 Discussion

The present study aimed to assess the prevalence of PTSD among cancer patients in Algeria and to validate the Arabic DSM‐5 PTSD Checklist (i.e., PCL‐5) among this population. Additionally, the study explored the association between demographic and clinical factors and the presence of PTSD. The findings indicated a range of PTSD symptoms among Algerian cancer patients, with 216 participants (58.4%) scoring above the threshold, suggesting a likelihood of PTSD. Moreover, CFA confirmed the structural validity of the PCL‐5. Key factors that were associated with PTSD included lower educational level and being in advanced stages of the disease. However, this suggests very limited clinical relevance because the symptoms of PTSD can vary significantly depending on coping mechanisms, social support systems, and other psychological variables such as levels of rumination, prior trauma, personality traits, and resilience [56, 57]. Research has shown that perceived social support helps buffer against PTSD symptoms, particularly numbing and re‐experiencing symptoms, while factors such as hope and resilience also play mediating roles [58, 59]. Additionally, a lack of positive social support and detrimental interactions has been associated with greater psychological comorbidity among cancer patients [58, 59, 60, 61]. While factors such as lower education levels and advanced disease stages were noted, their significance should not be overstated, because association does not imply causation. Future studies utilizing longitudinal designs would better approximate the dynamic relationship between these factors and PTSD among cancer patients, testing, if possible, interventions that may mitigate the psychological impact of diagnosis and treatment.

The present study provides valuable insights into the spectrum of PTSD symptoms among Algerian cancer patients, aligning with the broader literature on cancer‐related PTSD. Similar to findings from prior research [8, 15, 16, 48], which documented varying PTSD rates using different assessment methods, the present study also identified a range of symptom severities among cancer patients. Previous studies have reported PTSD prevalence up to 55% in specific populations [8], highlighting the significance of the present study's findings.

Cultural and contextual factors specific to Algerian cancer patients likely contribute to the observed symptomatology, including cultural beliefs, societal attitudes towards illness, and healthcare system structures [8]. By emphasizing nuanced PTSD symptom intensities among Algerian cancer patients, predominantly characterized by moderate to low distress levels, the present study extends understanding beyond clinical diagnostic thresholds. This approach contrasts with studies focusing solely on higher symptom severity or clinical diagnoses, thereby offering complementary insights into the heterogeneity of PTSD experiences within cancer populations [8, 21]. Ultimately, these findings underscore the necessity for culturally sensitive assessment and intervention strategies tailored to the unique needs of Algerian cancer patients. Further research exploring the interplay between cultural factors, assessment methodologies, and PTSD symptomatology is essential for advancing a comprehensive understanding of diverse cancer patient populations and enhancing global clinical practices. Developing assessment tools capable of accurately capturing the unique experiences of this population allows for a comprehensive assessment of PTSD symptomatology among cancer patients, facilitating better and earlier healthcare planning and the design of effective health promotion interventions in diverse contexts.

The results of the fit indices in the CFA supported the four‐factor structure for the PCL‐5. All loadings were above 0.5, except for Items 4 and 5. Item 4 (loading = 0.28), *“Feeling very upset when something reminded you of the stressful experience”* and Item 5 (loading = 0.26), *“Having strong physical reactions when something reminded you of the stressful experience”*. This might be due to cultural differences in how emotional and physical reactions to stress are experienced and reported. Despite this, these items may still provide valuable information about specific aspects of PTSD symptoms within this population.

Previous studies have established the validity and reliability of the PCL‐5 across diverse cultural contexts [25, 26, 27]. In the Arabic context, the PCL‐5 has proven effective for PTSD identification among Kurdish and Arab displaced populations [28]. In the Algerian context, where similar socio‐cultural factors (e.g., Islamic values, the emphasis on family and community ties, and shared Arab identity) may influence PTSD prevalence and expression, the PCL‐5's proven reliability in various settings underscores its potential utility and effectiveness in assessing PTSD symptoms.

The present study's findings indicated that higher educational attainment was associated with lower PTSD scores, suggesting that education may serve as a protective factor against the development or severity of PTSD symptoms among this population. Specifically, individuals with primary education had the highest PTSD mean score of 42.04. In contrast, those with secondary and university education reported significantly lower scores, reinforcing that educational level may influence psychological resilience. This aligns with prior research demonstrating similar protective effects of education in various trauma‐exposed populations [35, 36]. Conversely, the present study found a positive association between advanced disease stages at diagnosis and increased PTSD severity. This aligns with the observed differences in PTSD scores by disease stage: individuals in the first stage had the lowest mean score (33.59), while those in the second stage exhibited a markedly higher mean score (40.23), echoing previous findings that disease progression significantly impacts psychological outcomes among cancer patients [36]. Additionally, the analysis highlighted significant gender differences in PTSD scores, with males scoring higher (*M* = 38.66) than females (*M* = 34.80), indicating that gender may also play a role in PTSD vulnerability.

Contrary to some previous studies, the present study did not find significant associations between economic status or marital status and PTSD scores among cancer patients. For instance, Stuber et al. [34] reported lower income and being unmarried as risk factors for PTSD among cancer survivors. Such discrepancies may reflect differences in sample characteristics, cultural contexts, or the operationalization of variables across studies. Similarly, while treatment‐related factors such as surgery and chemotherapy duration were not significant predictors in the present study, the literature offers mixed evidence on their impact on PTSD, underscoring the need for further investigation into these variables' nuanced effects [35, 36].

Overall, the present study's findings emphasized the multifaceted nature of PTSD among cancer patients, highlighting how demographic and clinical variables interact in complex ways to influence psychological outcomes. Future research should not only aim to replicate these findings across diverse populations but also explore additional factors, such as social support and coping strategies, that may mediate the relationship between cancer‐related experiences and PTSD risk. Importantly, investigating the impact of psychological support interventions on PTSD levels is crucial. Effective psychological support can potentially mitigate PTSD symptoms and enhance the overall quality of life for cancer survivors. Insights from such studies will be essential for developing targeted interventions that address the unique needs of this population.

### 4.1 Theoretical Implications

The present study enhances the theoretical understanding of PTSD in the context of cancer among Algerian patients, contributing to the existing literature on PTSD across diverse populations. By validating the Arabic version of the DSM‐5 PTSD Checklist (PCL‐5) for this group, the study demonstrates its applicability across different cultural contexts, affirming its reliability as an assessment tool. This validation is crucial for broadening theoretical frameworks that explore how PTSD manifests in varied cultural and demographic settings. Additionally, identifying mild to moderate PTSD symptoms provides a nuanced perspective that goes beyond traditional clinical thresholds, offering insights into how cultural beliefs, societal views on illness, and the healthcare system in Algeria shape PTSD experiences. This understanding refines theories regarding the impact of socio‐cultural factors on PTSD severity and expression. Furthermore, the finding that higher educational attainment protects against PTSD development aligns with theoretical models linking education to resilience among trauma‐exposed populations [62, 63]. The observed correlation between advanced disease stages and increased PTSD severity also supports propositions regarding the influence of clinical variables on psychological distress. These findings highlight the need for tailored psychological interventions that consider the clinical stages of cancer.

### 4.2 Clinical Implications

The study offers essential insights for enhancing PTSD management among Algerian cancer patients, emphasizing both clinical and practical applications. Validating the Arabic DSM‐5 PTSD Checklist (PCL‐5) provides clinicians with a reliable tool for identifying PTSD symptoms early, which is crucial for timely and targeted interventions. Patients with lower educational levels and those in advanced cancer stages show higher vulnerability to PTSD, highlighting the need for prioritized psychological support. Educational programs that enhance mental health awareness and coping skills may be especially beneficial for patients with limited education, potentially reducing PTSD risks. Additionally, oncology care for patients in advanced stages should include frequent psychological assessments and customized interventions, because these individuals are at greater risk of severe PTSD symptoms.

The findings also underscore the importance of culturally sensitive assessment protocols, given that sociocultural factors—such as beliefs about illness and stigma around mental health—can significantly influence PTSD expression. Implementing culturally informed psychoeducational sessions can help patients and their families understand the psychological impact of cancer, reducing stigma and promoting acceptance of mental health support. Although economic and marital status were not significantly associated with PTSD, it remains essential for healthcare providers to consider these and other psychosocial factors in a comprehensive care approach. By integrating these insights into clinical practice, Algerian healthcare providers can better support the mental well‐being of cancer patients, improving their overall quality of life and treatment outcomes.

### 4.3 Limitations

Several limitations should be considered when interpreting the present study's findings. First, the cross‐sectional design limits causal inference regarding the relationship between demographic/clinical factors and PTSD. Longitudinal studies are needed to explore the dynamic nature of PTSD symptoms over time among Algerian cancer patients. Second, reliance on self‐report measures, common in PTSD research, may introduce response bias or underreporting of symptoms due to cultural factors influencing symptom disclosure. Future research should incorporate multi‐method approaches to enhance the validity of PTSD assessments among diverse populations. Third, the study's generalizability and estimation of prevalence may be constrained by its specific sample characteristics. Replication in larger and more diverse samples across Algeria would strengthen the external validity of the findings.

Fourth, a key limitation of this study was the exclusion of other psychological and clinical variables that could have influenced the presence and severity of PTSD. Variables such as levels of rumination, history of prior trauma, personality traits, resilience, and other comorbid psychological disorders (e.g., anxiety or depression) were not examined. These factors have been shown in prior research to play critical roles in the development and persistence of PTSD symptoms [58, 59, 60, 61]. Future studies should adopt a more comprehensive approach by including these variables to better understand the multifactorial nature of PTSD and its underlying mechanisms. In addition, the present study did not assess types of cancer. Therefore, the psychosocial impacts regarding the type of cancer cannot be assessed. More specifically, different types of cancer may have different levels of psychosocial distress due to their specific features. For example, those with head and neck cancer would have obvious appearance and body image deficits and might have worse psychological health than those with cancers showing no appearance deficits. Therefore, future studies are needed to examine the role of different types of cancer in PTSD.

## 5 Conclusion

The PCL‐5 demonstrated good psychometric properties, including internal consistency and structural validity, making it an appropriate tool for assessing PTSD symptoms among Algerian cancer patients. The study contributes valuable insights into PTSD among Algerian cancer patients, emphasizing the complexity of demographic and clinical factors influencing PTSD severity. The validated PCL‐5 highlights the protective role of education, underscoring its practical implications for clinical practice in assessing and managing PTSD symptoms. Future research should continue to explore additional factors influencing PTSD outcomes among diverse cancer patient populations, informing targeted interventions to improve psychological well‐being and quality of life for cancer survivors in Algeria.

## Ethics Statement

Ethical approval for this study was obtained from two institutions: Université Hassiba Benbouali de Chlef, Faculty of Humanities and Social Sciences (Approval No. 441/2022), and the University Hospital of Oran, Directorate of Medical and Paramedical Activities (Approval No. 234/2022/DAMP). The authorization was granted on May 30, 2022, by the University Hospital of Oran. The study was conducted in accordance with the Declaration of Helsinki and its subsequent amendments.

## Consent

Informed consent was obtained from all participants involved in the study.

## Conflicts of Interest

The authors declare no conflicts of interest.

## Data Availability

The dataset supporting this study’s findings is not openly available but is available from the corresponding author upon reasonable request.

## References

[1] M. Aljurf , N. S. Majhail , M. B. C. Koh , M. A. Kharfan‐Dabaja , and N. J. Chao , “Introduction,” in The Comprehensive Cancer Center: Development, Integration, and Implementation, eds. M. Aljurf , N. S. Majhail , M. B. C. Koh , M. A. Kharfan‐Dabaja , and N. J. Chao (Switzerland: Springer, 2022), 1–2, 10.1007/978-3-030-82052-7_1. 36121970

[2] F. Bray and K. D. Shield , “Cancer: Global Burden, Trends, and Projections,” in International Encyclopedia of Public Health, ed. S. R. Quah . 2nd ed. (Oxford, NY: Academic Press, 2017), 347–368, 10.1016/B978-0-12-803678-5.00052-7.

[3] R. L. Siegel , K. D. Miller , N. S. Wagle , and A. Jemal , “Cancer Statistics, 2023,” CA: A Cancer Journal for Clinicians 73, no. 1 (2023): 17–48, 10.3322/caac.21763. 36633525

[4] F. Bray , M. Laversanne , H. Sung , et al., “Global Cancer Statistics 2022: GLOBOCAN Estimates of Incidence and Mortality Worldwide for 36 Cancers in 185 Countries,” CA: A Cancer Journal for Clinicians 74, no. 3 (2024): 229–263, 10.3322/caac.21834. 38572751

[5] S. Shrivastava , P. Shrivastava , and J. Ramasamy , “World Health Organization Emphasizes for the Development of the Priority List of Medical Devices for the Management of Cancers,” International Journal of Health & Allied Sciences 6, no. 4 (2017): 245, 10.4103/ijhas.ijhas_90_17.

[6] T. P. Kingham and S. L. Wong , “Global Surgical Oncology: Addressing the Global Surgical Oncology Disease Burden,” Annals of Surgical Oncology 22, no. 3 (2015): 708–709, 10.1245/s10434-014-4347-5. 25572683

[7] G. W. Prager , S. Braga , B. Bystricky , et al., “Global Cancer Control: Responding to the Growing Burden, Rising Costs, and Inequalities in Access,” ESMO Open 3, no. 2 (2018): e000285, 10.1136/esmoopen-2017-000285. 29464109 PMC5812392

[8] G. Abbey , S. B. N. Thompson , T. Hickish , and D. Heathcote , “A Meta‐Analysis of Prevalence Rates and Moderating Factors for Cancer‐Related Post‐Traumatic Stress Disorder,” Psycho‐Oncology 24, no. 4 (2015): 371–381, 10.1002/pon.3654. 25146298 PMC4409098

[9] É. Kállay and C. L. Dégi , “Distress in Cancer Patients,” Cognitie, Creier, Comportament/Cognition, Brain, Behavior 18, no. 1 (2014): 17–38.

[10] S. Vehling and D. W. Kissane , “Existential Distress in Cancer: Alleviating Suffering From Fundamental Loss and Change,” Psycho‐Oncology 27, no. 11 (2018): 2525–2530, 10.1002/pon.4872. 30307088

[11] C. M. H. Chan , C. G. Ng , N. A. Taib , L. H. Wee , E. Krupat , and F. Meyer , “Course and Predictors of Post‐Traumatic Stress Disorder in a Cohort of Psychologically Distressed Patients With Cancer: A 4‐Year Follow‐Up Study,” Cancer 124, no. 2 (2018): 406–416, 10.1002/cncr.30980. 29152719

[12] X. Wu , J. Wang , R. Cofie , A. C. Kaminga , and A. Liu , “Prevalence of Posttraumatic Stress Disorder Among Breast Cancer Patients: A Meta‐Analysis,” Iranian Journal of Public Health 45, no. 12 (2016): 1533–1544. 28053919 PMC5207094

[13] M. A. Pereira , A. Araújo , M. Simões , and C. Costa , “Influence of Psychological Factors in Breast and Lung Cancer Risk—A Systematic Review,” Frontiers in Psychology 12 (2022): 769394, 10.3389/fpsyg.2021.769394. 35046872 PMC8762112

[14] M. R. Widows , P. B. Jacobsen , M. Booth‐Jones , and K. K. Fields , “Predictors of Posttraumatic Growth Following Bone Marrow Transplantation for Cancer,” Health Psychology 24, no. 3 (2005): 266–273, 10.1037/0278-6133.24.3.266. 15898862

[15] M. J. Cordova , M. B. Riba , and D. Spiegel , “Post‐Traumatic Stress Disorder and Cancer,” Lancet Psychiatry 4, no. 4 (2017): 330–338, 10.1016/S2215-0366(17)30014-7. 28109647 PMC5676567

[16] M. Kangas , “DSM‐5 Trauma and Stress‐Related Disorders: Implications for Screening for Cancer‐Related Stress,” Frontiers in Psychiatry 4 (2013): 122, 10.3389/fpsyt.2013.00122. 24106482 PMC3788331

[17] G. Martino , A. Catalano , R. M. Agostino , et al., “Quality of Life and Psychological Functioning in Postmenopausal Women Undergoing Aromatase Inhibitor Treatment for Early Breast Cancer,” PLoS One 15, no. 3 (2020): e0230681, 10.1371/journal.pone.0230681. 32214378 PMC7098625

[18] M. Y. Smith , W. H. Redd , C. Peyser , and D. Vogl , “Post‐Traumatic Stress Disorder in Cancer: A Review.” Psycho‐Oncology 8, no. 6 (1999): 521–537, 10.1002/(sici)1099-1611(199911/12)8:6<521::aid-pon423>3.0.co;2-x. 10607985

[19] B. D. Bultz and L. E. Carlson , “Emotional Distress: The Sixth Vital Sign—Future Directions in Cancer Care,” Psycho‐Oncology 15, no. 2 (2006): 93–95, 10.1002/pon.1022. 16444764

[20] L. Fallowfield and V. Jenkins , “Communicating Sad, Bad, and Difficult News in Medicine,” Lancet 363, no. 9405 (2004): 312–319, 10.1016/S0140-6736(03)15392-5. 14751707

[21] C. Carmassi , V. Pedrinelli , S. Fantasia , et al., “Post‐Traumatic Stress and Depressive Symptoms in Women With Ovarian Cancer 3–6 Months After Diagnosis,” Anticancer Research 44, no. 2 (2024): 829–838, 10.21873/anticanres.16875. 38307582

[22] M. Unseld , K. Krammer , S. Lubowitzki , et al., “Screening for Post‐Traumatic Stress Disorders in 1017 Cancer Patients and Correlation With Anxiety, Depression, and Distress,” Psycho‐Oncology 28, no. 12 (2019): 2382–2388, 10.1002/pon.5239. 31679172 PMC6916606

[23] M. Doolittle and K. N. DuHamel , “Posttraumatic Stress Disorder Associated With Cancer Diagnosis and Treatment,” in Psycho‐Oncology, eds. W. S. Breitbart , W. Breitbart , P. Butow , et al. (New York: Oxford University Press, 2021), 363–373, 10.1093/med/9780190097653.003.0047.

[24] A. Leano , M. B. Korman , L. Goldberg , and J. Ellis , “Are We Missing PTSD in Our Patients With Cancer? Part I,” Canadian Oncology Nursing Journal 29, no. 2 (2019): 141–146. 31148714 PMC6516338

[25] T. Carvalho , C. da Motta , and J. Pinto‐Gouveia , “Portuguese Version of the Posttraumatic Stress Disorder Checklist for DSM‐5 (PCL‐5): Comparison of Latent Models and Other Psychometric Analyses,” Journal of Clinical Psychology 76, no. 7 (2020): 1267–1282, 10.1002/jclp.22930. 31975500

[26] M. Ito , Y. Takebayashi , Y. Suzuki , and M. Horikoshi , “Posttraumatic Stress Disorder Checklist for DSM‐5: Psychometric Properties in a Japanese Population,” Journal of Affective Disorders 247 (2019): 11–19, 10.1016/j.jad.2018.12.086. 30640025

[27] D. L. G. Van Praag , H. E. Fardzadeh , A. Covic , A. I. R. Maas , and N. von Steinbüchel , “Preliminary Validation of the Dutch Version of the Posttraumatic Stress Disorder Checklist for DSM‐5 (PCL‐5) After Traumatic Brain Injury in a Civilian Population,” PLoS One 15, no. 4 (2020): e0231857, 10.1371/journal.pone.0231857. 32310970 PMC7170250

[28] H. Ibrahim , V. Ertl , C. Catani , A. A. Ismail , and F. Neuner , “The Validity of Posttraumatic Stress Disorder Checklist for DSM‐5 (PCL‐5) as Screening Instrument With Kurdish and Arab Displaced Populations Living in the Kurdistan Region of Iraq,” BMC Psychiatry 18, no. 1 (2018): 259, 10.1186/s12888-018-1839-z. 30115040 PMC6097219

[29] C. M. Hoeboer , I. Karaban , J. F. Karchoud , M. Olff , and M. van Zuiden , “Validation of the PCL‐5 in Dutch Trauma‐Exposed Adults,” BMC Psychology 12, no. 1 (2024): 456, 10.1186/s40359-024-01951-y. 39198929 PMC11351185

[30] M. Di Tella , A. Romeo , G. Zara , L. Castelli , and M. Settanni , “The Post‐Traumatic Stress Disorder Checklist for DSM‐5: Psychometric Properties of the Italian Version,” International Journal of Environmental Research and Public Health 19, no. 9 (2022): 5882, 10.3390/ijerph19095282. 35564677 PMC9105570

[31] Z. Z. Cernovsky , M. Fattahi , L. C. Litman , and D. M. Diamond , “Validation of the PTSD Checklist for DSM‐5 (PCL‐5) on Patients Injured in Car Accidents,” European Journal of Medical and Health Sciences 3, no. 2 (2021): 154–159, 10.24018/ejmed.2021.3.2.790.

[32] K. N. DuHamel , J. Ostrof , T. Ashman , et al., “Construct Validity of the Posttraumatic Stress Disorder Checklist in Cancer Survivors: Analyses Based on Two Samples,” Psychological Assessment 16, no. 3 (2004): 255–266, 10.1037/1040-3590.16.3.255. 15456381

[33] S. K. Smith , C. Manschot , E. Kuhn , et al., “Assessing the Utility of the PC‐PTSD‐5 as a Screening Tool Among a Cancer Survivor Sample,” Cancer 130, no. 23 (2024): 4118–4126, 10.1002/cncr.35504. 39141666 PMC11560558

[34] M. L. Stuber , K. A. Meeske , K. R. Krull , et al., “Prevalence and Predictors of Posttraumatic Stress Disorder in Adult Survivors of Childhood Cancer,” Pediatrics 125, no. 5 (2010): e1124–e1134, 10.1542/peds.2009-2308. 20435702 PMC3098501

[35] M. C. Chung and M. C. Chung , “Posttraumatic Stress Disorder and Cancer: Secondary Victims,” in Posttraumatic Stress in Physical Illness, ed. M. C. Chung (UK: Ashford Colour Press Ltd, 2024), 10.1093/oso/9780198727323.001.0001.

[36] M. Naderi , M. Firouzkoohi Moghadam , M. Hamzenejad , A. Emamdadi , and H. Karami , “Post‐Traumatic Stress Disorder and Related Factors in Parents of Children With Cancer in South‐East of Iran,” Iranian Red Crescent Medical Journal 14, no. 12 (2012): 776–781, 10.5812/ircmj.2163. 23483014 PMC3587866

[37] C. A. Blevins , F. W. Weathers , M. T. Davis , T. K. Witte , and J. L. Domino , “The Posttraumatic Stress Disorder Checklist for DSM‐5 (PCL‐5): Development and Initial Psychometric Evaluation,” Journal of Traumatic Stress 28, no. 6 (2015): 489–498, 10.1002/jts.22059. 26606250

[38] M. J. Bovin , B. P. Marx , F. W. Weathers , et al., “Psychometric Properties of the PTSD Checklist for Diagnostic and Statistical Manual of Mental Disorders‐Fifth Edition (PCL‐5) in Veterans,” Psychological Assessment 28, no. 11 (2016): 1379–1391, 10.1037/pas0000254. 26653052

[39] P. B. Marx , D. J. Lee , S. B. Norman , et al., “Reliable and Clinically Significant Change in the Clinician‐Administered PTSD Scale for DSM‐5 and PTSD Checklist for DSM‐5 Among Male Veterans,” Psychological Assessment 34, no. 2 (2022): 197–203, 10.1037/pas0001098. 34941354 PMC9022599

[40] National Center for PTSD in the U.S. Department of Veterans Affairs 2018. “PTSD Checklist for DSM‐5 (PCL‐5),” Retrieved January 12, 2025, https://www.ptsd.va.gov/professional/assessment/adult-sr/ptsd-checklist.asp.

[41] A. Krüger‐Gottschalk , C. Knaevelsrud , H. Rau , et al., “The German Version of the Posttraumatic Stress Disorder Checklist for DSM‐5 (PCL‐5): Psychometric Properties and Diagnostic Utility,” BMC Psychiatry 17, no. 1 (2017): 379, 10.1186/s12888-017-1541-6. 29183285 PMC5704375

[42] E. d. P. Lima , A. G. Vasconcelos , W. Berger , et al., “Cross‐Cultural Adaptation of the Posttraumatic Stress Disorder Checklist 5 (PCL‐5) and Life Events Checklist 5 (LEC‐5) for the Brazilian Context,” Trends in Psychiatry and Psychotherapy 38, no. 4 (2016): 207–215, 10.1590/2237-6089-2015-0074. 28076641

[43] M. Boysan , P. Guzel Ozdemir , O. Ozdemir , Y. Selvi , E. Yilmaz , and N. Kaya , “Psychometric Properties of the Turkish Version of the PTSD Checklist for Diagnostic and Statistical Manual of Mental Disorders, (PCL‐5),” Psychiatry and Clinical Psychopharmacology 27, no. 3 (2017): 300–310, 10.1080/24750573.2017.1342769.

[44] F. Alhalaiqa , O. A. Alfuqaha , R. Masa’Deh , et al., “Psychometric Properties of the Posttraumatic Stress Disorder Checklist Among the Lebanese Population Exposed to the Beirut Explosion: A Cross‐Sectional Study During the COVID‐19 Pandemic,” Behavioural Neurology 2023, no. 1 (2023): 9286562–9286569, 10.1155/2023/9286562. 37822368 PMC10564576

[45] A. Sabah , M. A. Aljaberi , K.‐H. Lee , and C.‐Y. Lin , “Psychometric Properties of the Perceived Collective Family Efficacy Scale in Algeria,” Healthcare 11, no. 19 (2023): 2691, 10.3390/healthcare11192691. 37830728 PMC10572840

[46] A. Sabah , O. Khalaf Rashid Al‐Shujairi , and S. Boumediene , “The Arabic Version of the Walsh Family Resilience Questionnaire: Confirmatory Factor Analysis of a Family Resilience Assessment Among Algerian and Iraq Families,” International Journal of Systemic Therapy 32, no. 4 (2021): 273–290, 10.1080/2692398X.2021.1960117.

[47] A. R. Ashbaugh , S. Houle‐Johnson , C. Herbert , W. El‐Hage , and A. Brunet , “Psychometric Validation of the English and French Versions of the Posttraumatic Stress Disorder Checklist for DSM‐5 (PCL‐5),” PLoS One 11, no. 10 (2016): e0161645, 10.1371/journal.pone.0161645. 27723815 PMC5056703

[48] A. Jung , J. L. Crandell , M. E. Nielsen , D. K. Mayer , and S. K. Smith , “Post‐Traumatic Stress Disorder Symptoms in Non‐Muscle‐Invasive Bladder Cancer Survivors: A Population‐Based Study,” Urologic Oncology: Seminars and Original Investigations 39, no. 4 (2021): 237.e237–237.e214, 10.1016/j.urolonc.2020.11.033. PMC7956138 33308978

[49] R. Verhey , D. Chibanda , L. Gibson , J. Brakarsh , and S. Seedat , “Validation of the Posttraumatic Stress Disorder Checklist–5 (PCL‐5) in a Primary Care Population With High HIV Prevalence in Zimbabwe,” BMC Psychiatry 18, no. 1 (2018): 109, 10.1186/s12888-018-1688-9. 29685117 PMC5913864

[50] M. A. Aljaberi , M. A. Al‐Sharafi , M. U. H. Uzir , et al., “Psychological Toll of the COVID‐19 Pandemic: An In‐Depth Exploration of Anxiety, Depression, and Insomnia and the Influence of Quarantine Measures on Daily Life,” Healthcare 11, no. 17 (2023): 2418, 10.3390/healthcare11172418. 37685451 PMC10487588

[51] M. A. Aljaberi , K.‐H. Lee , N. A. Alareqe , et al., “Rasch Modeling and Multilevel Confirmatory Factor Analysis for the Usability of the Impact of Event Scale‐Revised (IES‐R) During the COVID‐19 Pandemic,” Healthcare 10, no. 10 (2022): 1858, 10.3390/healthcare10101858. 36292305 PMC9602035

[52] A. Sabah , M. A. Aljaberi , J. Hajji , C.‐Y. Fang , Y.‐C. Lai , and C.‐Y. Lin , “Family Communication as a Mediator Between Family Resilience and Family Functioning Under the Quarantine and COVID‐19 Pandemic in Arabic Countries,” Children 10, no. 11 (2023): 1742, 10.3390/children10111742. 38002833 PMC10670761

[53] A. Sabah , M. A. Aljaberi , C.‐Y. Lin , and H.‐P. Chen , “The Associations Between Sibling Victimization, Sibling Bullying, Parental Acceptance–Rejection, and School Bullying,” International Journal of Environmental Research and Public Health 19, no. 23 (2022): 16346, 10.3390/ijerph192316346. 36498416 PMC9739229

[54] F. Z. E. Abiddine , M. A. Aljaberi , A. Alduais , C.‐Y. Lin , Z. Vally , and M. D. Griffiths , “The Psychometric Properties of the Arabic Bergen Social Media Addiction Scale,” International Journal of Mental Health and Addiction (2024): Advance online publication, 10.1007/s11469-024-01297-x.

[55] N. A. Alareqe , S. A. Hassan , E. M. E. Kamarudin , et al., “Validity of Adult Psychopathology Model Using Psychiatric Patient Sample From a Developing Country: Confirmatory Factor Analysis,” Mental Illness 2022, no. 1 (2022): 9594914–9595012, 10.1155/2022/9594914.

[56] K. Knauer , A. Bach , N. Schäffeler , A. Stengel , and J. Graf , “Personality Traits and Coping Strategies Relevant to Posttraumatic Growth in Patients With Cancer and Survivors: A Systematic Literature Review,” Current Oncology 29, no. 12 (2022): 9593–9612, 10.3390/curroncol29120754. 36547168 PMC9776882

[57] A. Seiler and J. Jenewein , “Resilience in Cancer Patients,” Frontiers in Psychiatry 10 (2019): 208, 10.3389/fpsyt.2019.00208. 31024362 PMC6460045

[58] C. Liu , Y. Zhang , H. Jiang , and H. Wu , “Association Between Social Support and Post‐Traumatic Stress Disorder Symptoms Among Chinese Patients With Ovarian Cancer: A Multiple Mediation Model,” PLoS One 12, no. 5 (2017): e0177055, 10.1371/journal.pone.0177055. 28475593 PMC5419605

[59] C. L. Liu , L. Liu , Y. Zhang , X. Z. Dai , and H. Wu , “Prevalence and Its Associated Psychological Variables of Symptoms of Depression and Anxiety Among Ovarian Cancer Patients in China: A Cross‐Sectional Study,” Health and Quality of Life Outcomes 15, no. 1 (2017): 161, 10.1186/s12955-017-0738-1. 28818112 PMC5561632

[60] G. Costa‐Requena , R. Ballester Arnal , and F. Gil , “The Influence of Coping Response and Health‐Related Quality of Life on Perceived Social Support During Cancer Treatment,” Palliative & Supportive Care 13, no. 3 (2015): 683–689, 10.1017/S1478951514000418. 24774413

[61] A. Mehnert , C. Lehmann , M. Graefen , H. Huland , and U. Koch , “Depression, Anxiety, Post‐Traumatic Stress Disorder and Health‐Related Quality of Life and Its Association With Social Support in Ambulatory Prostate Cancer Patients,” European Journal of Cancer Care 19, no. 6 (2010): 736–745, 10.1111/j.1365-2354.2009.01117.x. 19832893

[62] S. B. Morissette , C. Ryan‐Gonzalez , T. Yufik , et al., “The Effects of Posttraumatic Stress Disorder Symptoms on Educational Functioning in Student Veterans,” Psychological Services 18, no. 1 (2021): 124–133, 10.1037/ser0000356. 31192672 PMC7003209

[63] A. Vilaplana‐Pérez , A. Sidorchuk , A. Pérez‐Vigil , et al., “Assessment of Posttraumatic Stress Disorder and Educational Achievement in Sweden,” JAMA Network Open 3, no. 12 (2020): e2028477, 10.1001/jamanetworkopen.2020.28477. 33289847 PMC7724559

